# OA09.02. Observation on effects of 10.6µm laser moxibustion in patients with knee osteoarthritis: a double-blind, randomized, controlled study

**DOI:** 10.1186/1472-6882-12-S1-O34

**Published:** 2012-06-12

**Authors:** F Wu, L Zhao, W Lizhen, H Zhang, L Lao, X Shen

**Affiliations:** 1 Shanghai University of Traditional Chinese Medicine, Shanghai, China; 2 University of Maryland School of Medicine, Baltimore, USA

## Purpose

To observe whether 10.6μm laser moxibustion provides greater pain relief, improved joint stiffness and function in comparison with sham laser moxibustion.

## Methods

172 patients with knee osteoarthritis were randomly divided into real and sham laser moxibustion groups, with 10.6μm laser moxibustion and sham laser moxibustion treatment on ST-35 respectively. Patients in both groups received 20 minutes of treatment, thrice a week and 4 weeks in total. Effects of treatment were assessed mainly by changes in the Western Ontario and McMaster Universities Osteoarthritis Index (WOMAC VA 3.1) before, in the middle (after 2 weeks), at the end (after 4 weeks) and 4 weeks after the end of the treatment. Completion time of 50 yards walking was evaluated as a secondary measurement.

## Results

There was no statistical difference in WOMAC pain, stiffness and function scores between the two groups before treatment. Patients in the real treatment group experienced greater improvement in WOMAC pain, stiffness and function scores in the middle, at the end and 4 weeks after the end of the treatment (p<0.05). No significant difference was shown in completion time of 50 yards walking before, in the middle and at the end of the treatment. Patients in the real treatment group were superior to those in the sham group in completion time of 50 yards’ walking 4 weeks after the treatment.

## Conclusion

Compared with sham treatment, 10.6μm laser moxibustion can significantly reduce pain and improve knee joint stiffness and function in patients with knee osteoarthritis.

